# Cobalamin (Vitamin B12) Induced a Shift in Microbial Composition and Metabolic Activity in an *in vitro* Colon Simulation

**DOI:** 10.3389/fmicb.2018.02780

**Published:** 2018-11-16

**Authors:** Yuanyuan Xu, Shasha Xiang, Kun Ye, Yiqing Zheng, Xiao Feng, Xuan Zhu, Jie Chen, Yuewen Chen

**Affiliations:** School of Food Science and Biotechnology, Zhejiang Gongshang University, Hangzhou, China

**Keywords:** cyanocobalamin, methylcobalamin, *in vitro* colon simulation, gut microbiota, *Acinetobacter*

## Abstract

Cobalamin deficiency is believed to be related to disturbances in cell division, neuropathy, nervous system disease and pernicious anemia. Elderly people are usually advised to supplement their diets with cobalamin. As cobalamin has several forms, the effects of different forms of cobalamin on gut microbiota were investigated in this study. After 7 days of supplementation, methylcobalamin had reduced the diversity of gut microbiota compared to that in the control and cyanocobalamin groups. After supplementation with methylcobalamin, the percentage of *Acinetobacter* spp. had increased to 45.54%, while the percentages of *Bacteroides* spp., *Enterobacteriaceae* spp. and *Ruminococcaceae* spp. had declined to 11.15, 9.34, and 2.69%, respectively. However, cyanocobalamin had different influences on these bacteria. Both cobalamins increased the amount of short-chain fatty acids, particularly butyrate and propionic acid. The cyanocobalamin group showed increased activity of cellulase compared with that in the other two groups. According to CCA and PICRUSt analysis, methylcobalamin had a positive correlation with *Pseudomonas* bacteria, propionic acid, and butyrate. Methylcobalamin promoted lipid, terpenoid, and polyketide metabolism by gut bacteria, promoted the degradation of exogenous substances, and inhibited the synthesis of transcription factors and secondary metabolites. Our results indicate that the various forms of cobalamin in the food industry should be monitored and regulated, because the two types of cobalamin had different effects on the gut microbiome and on microbial metabolism, although they have equal bio-activity in humans. Given the effects of methylcobalamin on gut microbiota and microbial metabolism, methylcobalamin supplementation should be suggested as the first option.

## Introduction

The human colon is the most important colonization location for gut microbiota, which reaches concentrations of 10^11^–10^12^ cells/g (Eckburg et al., 2005). There are various bacteria living in the colon, which are dominated by species in *Bacteroides*, *Bifidobacterium*, *Clostridium*, *Streptococcus*, *Eubacterium*, and other genera. Most are obligate anaerobes, and the rest are facultative anaerobes (Eckburg et al., 2005). One of the essential roles of gut microbiota is vitamin production. Many recent studies have indicated that gut microbiota are associated with nutrient digestion and absorption, immune reactions and obesity (Yu and Schwabe, 2017). Due to these functions, gut microbiota are considered vital factors in health and disease.

Cobalamin (vitamin B12) exists as methylcobalamin, cyanocobalamin, and other analogs that can be synthesized only by bacteria, archaebacteria, and limited algal species. Cobalamin deficiency is believed to be related to disturbances in cell division, neuropathy, nervous system disease, and pernicious anemia (Issac et al., 2015). High concentrations of cobalamin can be detected in fermented food. As plant-based food does not contain cobalamin, cobalamin deficiency is prevalent in vegetarians (Roth et al., 1996). The glycoprotein intrinsic factor (IF) secreted by the gastric mucosa plays an important role in the absorption of cobalamin. Due to deficiency of the intrinsic factor, some patients still lack cobalamin even though they obtain sufficient cobalamin through supplementation. Methylcobalamin tablets have been used as a prescription medicine to cure peripheral neuropathy. In that case, excess cobalamin supplement can enter the intestine to change the gut microbiome composition (Giannella et al., 1971).

Degnan et al. (2014) found that cobalamin can act as a regulator of the gut micro-community. They found that competition and exchange of cobalamin among microbes may change the microbial composition and functions. Giannella et al. (1971) demonstrated a relationship between cobalamin and intestinal bacterial overgrowth syndromes. Upon overgrowth of bacteria in the small intestine, the bacteria competitively take up cobalamin with IF, leading to cobalamin malabsorption. Cordonnier et al. (2016) found that *Bacteroides* spp. restricted the Shiga-like toxin gene expression of enterohemorrhagic *Escherichia coli* by absorbing cobalamin. Some researchers have also demonstrated the effects of cobalamin on gut microbiota in animal nutrition (Chen et al., 2007, 2012). However, there are great differences in gut microbiota in various animal species and in humans with different ages, diets, locations and other characteristics (Wang et al., 2015).

Thus far, there have been few reports on the effects of cobalamin on human gut microbiota. Therefore, the aim of this study was to investigate the effects of methyl- and cyanocobalamin on the gut microbiota and on metabolite shifts in cobalamin deficiency patients in an *in vitro* colon simulation. Based on this study, a suitable suggestion for cobalamin application in the food industry can be offered.

## Materials and Methods

### Fecal Sample Collection and Pre-treatment

Fecal samples were voluntarily donated by five cobalamin deficiency patients who did not receive antibiotics for at least 6 months and who signed the informed consent forms. Fresh fecal samples (10 g) were collected and dissolved in 90 mL of sterile PBS (0.1 mol/L, pH 4.7). Then, the samples were stirred thoroughly and filtered through three layers of gauze to discard large-grain food residue. The filtrates (bacterial suspension) were collected as the starting cultures for *in vitro* colon simulation.

### *In vitro* Colon Simulation

**FIGURE 1:**
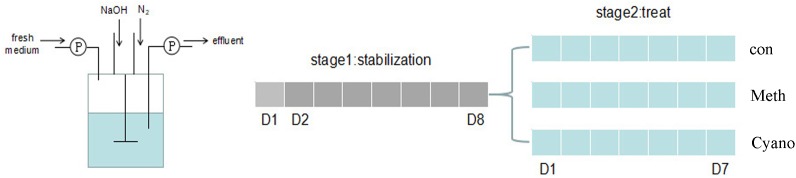
A diagram of *in vitro colonic* simulation and experiment design (Changdao Moni simulation). Gray: stabilization stage for the first 8 days. Blue: the experiment stages with/without Meth, and Cyano supplementation for 7 days.

*In vitro* colon fermentation was conducted in Changdao Moni simulation system (CDMNS) at Zhejiang Gongshang University (Figure 1). The ratio of the bacterial suspension to the nutritive medium was 1:9 (v/v). The nutrient medium consisted of (g/L) (Hangwei, Hangzhou, China): peptone 3.0; corn starch 8.0; yeast extract 4.5; tryptone 3.0; mucin 0.5; L-cysteine hydrochloride 0.8; bile salts no. 3 0.4; haem 0.05; sodium chloride 4.5; Tween 80 1.0; potassium chloride 2.5; potassium dihydrogen phosphate 0.4; magnesium chloride hexahydrate 4.5; calcium chloride hexahydrate 0.2; magnesium sulfate heptahydrate 3.0; ferrous sulfate heptahydrate 0.1; calcium chloride dihydrate 0.1; manganese chloride tetrahydrate 0.32; cobalt sulfate heptahydrate 0.18; copper sulfate pentahydrate 0.01; zinc sulfate heptahydrate 0.18; and nickel chloride hexahydrate 0.092 (Yin et al., 2013). The total volume of each reaction was 900 mL (stage 1).

Aliquots (90 mL) of starting cultures were added into the colon simulation with nutrient medium for 24 h. Nutrient medium was added into the simulation at a rate of 37.5 mL/h beginning on the 2nd day. The system became stable on the 8th day. After the first culture stage, 900 mL of fermentation broth was evenly distributed into reaction a, which was used as a control group; reaction b, which was supplemented with methylcobalamin; and reaction c, which was supplemented with cyanocobalamin (300 mL each) for the 2nd stage simulation. Methylcobalamin and cyanocobalamin (1.25 mg/L) were added into the nutrient medium for reactions b (methylcobalamin) and c (cyanocobalamin), respectively. The control group (*Con*), the methylcobalamin group (*Meth*), and the cyanocobalamin group (*Cyano*) were used to investigate shifts in the gut microbiota under various cobalamin conditions. The experiments lasted for another 7 days. During the entire fermentation process, the speed of the stirring rotor was set at 80 r/min. All the systems were kept at 37°C. The pH values were maintained at 6.8 by the addition of 0.5 mol/L NaOH and HCl when necessary. Anaerobic conditions were generated by flushing the headspace of all reactions and medium vessels with N_2_ for 30 min three times per day. 20 mL of fermentation broth samples were collected every evening and stored at 4°C. The experiments were conducted in triplicate.

### Short-Chain Fatty Acid Analysis

Gas chromatography–mass spectrometry (GC-MS) analysis was performed to determine the relative levels of short-chain fatty acids (SCFAs) according to the methods of Tan et al. (2005) with modification. Batch cultures (10 mL) were harvested and centrifuged at 8000 r/min for 2 min. The supernatant was collected and filtered through a 0.45 μm filter membrane. Sulfuric acid solution (50%; 0.1 mL) and 1.0 mL of ethyl ether were added to the 0.5 mL treatment suspension. The mixture was shaken 30 times, centrifuged at 10000 r/min for 5 min, and stored at 4°C for 30 min. The solution was then subjected to GC-MS analysis. All of the analyses were performed at room temperature, and each sample was placed on a FFAP elastic quartz capillary column (30 m × 0.25 mm × 0.25 μm). All parameters were set as follows: oven temperature: increasing from 100°C (1 min) to 150°C (5 min) at a rate of 5°C per min; source temperature: 200°C; interface temperature: 250°C; carrier gas: high-purity nitrogen (≥99.999%); carrier gas flow rate: 2 mL/min; inlet temperature: 270°C; injection method: splitless injection; injection volume: 2.0 μL; detector temperature (FID): 280°C; mass spectrometer ion source type: electrospray ionization (ESI); ion source temperature: 350°C.

### Enzyme Activity Measurement

For enzymatic analysis, samples of fermentation broth were analyzed under aseptic conditions. The fermentation broth samples were shaken at 20 r/min for 30 min on a rotating shaker and centrifuged at 4000 r/min for 5 min. The suspension was centrifuged at 4000 ×*g* for 5 min. After centrifugation, the supernatant was collected to measure enzyme activity. Cellulase activity was measured by the 3,5-dinitrosalicylic acid (DNS) method (Gusakov et al., 2011). Amylase activity was evaluated by the iodine-starch colorimetric method (Shi et al., 2015). Protease activity was determined by the Folin phenol reagent method (Shi et al., 2015). In this study, one amylase activity unit was defined as the quantity of enzyme in 1 mL of enzyme solution needed to degrade 10 mg of starch in 30 min at 40°C. One protease activity unit was defined as the quantity of enzyme in 1 mL of enzyme solution needed to cleave casein and produce 1 μg of tyrosine per minute at 40°C at a pH value of 7.0. One unit of cellulase activity was defined as the quantity of enzyme in 1 mL of enzyme solution needed to hydrolyse carboxymethyl cellulose to 1 mg of glucose in 30 min.

### DNA Isolation, PCR, and 16S rRNA Gene Analysis

DNA from different samples was extracted using a Genomic DNA Kit (Qiagen, Hilden, Germany) according to the manufacturer’s protocol. Total DNA was eluted in 50 μL of elution buffer by a modified version of the procedure described by the manufacturer (Qiagen, Hilden, Germany), and the samples were stored at -80°C until PCR amplification (LC-Bio Technology Co., Ltd., Hangzhou, Zhejiang Province, China). Bacterial 16S rRNA gene sequences (V3-V4 regions) were amplified from the whole genome of the samples via the primers 319F (5′-ACTCCTACGGGAGGCAGCAG-3′) and 806R (5′-GGACTACHVGGGTWTCTAAT-3′) according to a previous method with a small modification (Huang et al., 2016). All reactions were conducted in 25 μL total volumes including approximately 25 ng of genomic DNA extract, 12.5 μL of PCR premix, 2.5 μL of each primer, and PCR-grade water to adjust the volume. PCR was performed in a MasterCycler Gradient thermal cycler (Eppendorf, Hamburg, Germany) set to the following conditions: initial denaturation at 98°C for 30 s; 35 cycles of denaturation at 98°C for 10 s, annealing at 54/52°C for 30 s, and extension at 72°C for 45 s; and final extension at 72°C for 10 min.

The PCR products were normalized by an AxyPrepTM Mag PCR Normalizer (Axygen Biosciences, Union City, CA, United States), which allowed skipping the quantification step regardless of the PCR volume submitted for sequencing. The amplicon pools were prepared for sequencing with AMPure XT beads (Beckman Coulter Genomics, Danvers, MA, United States), and the size and quantity of the amplicon library were assessed on the LabChip GX (Perkin Elmer, Waltham, MA, United States) and with the Library Quantification Kit for Illumina (Kapa Biosciences, Woburn, MA, United States), respectively. The PhiX Control library (V3) (Illumina) was combined with the amplicon library (expected at 30%). The library was clustered to a density of approximately 570 K/mm^2^. The libraries were sequenced on the 300PE MiSeq runs, and one library was sequenced with both protocols using the standard Illumina sequencing primers, eliminating the need for a third (or fourth) index read.

16S rRNA gene sequences were processed and modified as in previously described methods (Zhang et al., 2013). The reads were controlled and confirmed with QIIME (Quantitative Insights Into Microbial Ecology^1^), VSEARCH^2^, FastQC, and FLASH (Fast Length Adjustment of SHort reads, v1.2.8^3^) quality filters. The CD-HIT method was used to select operational taxonomic units (OTUs) by generation of an OTU table (Li and Godzik, 2006). When the similarity of OTUs was over 97%, the sequences were assigned to one unit. RDP (Ribosomal Database Project) classifiers were applied to distribute the 16S rRNA genes into distinct taxonomic categories by aligning representative sequences to taxonomically annotated sequences (Ghodsi et al., 2011). To calculate the alpha diversity, we rarefied the OTU table and calculated two metrics. The Chao1 and Shannon indices are two commonly used alpha diversity indices (Zhang et al., 2013). The Chao1 index reflects the estimated number of OTU species in the sample. The Shannon index mainly reflects the average or uniformity of the abundance of different species in the sample. A heatmap of an important family of gut microbes was created with MeV 4.9.0. PICRUSt software was used to predict the gene functions for the OTU tables^4^. Principal component analysis (PCA) and canonical correlation analysis (CCA) were performed for all the taxa relative abundances of the 16S rDNA sequencing results via MATLAB software (v8.1.0.430).

### Statistics

All data are shown as the means ± SD. The data were analyzed with ANOVA and Duncan’s test for multiple comparisons with SPSS ver. 17.0. A value of *p* < 0.05 was considered significant.

### Data Access

The 16S sequence data generated in this study have been submitted to the NCBI Sequence Read Archive under accession number SRP151050.

## Results

### Effects of Methylcobalamin (*Meth*) and Cyanocobalamin (*Cyano*) on the Bacterial Community

**FIGURE 2:**
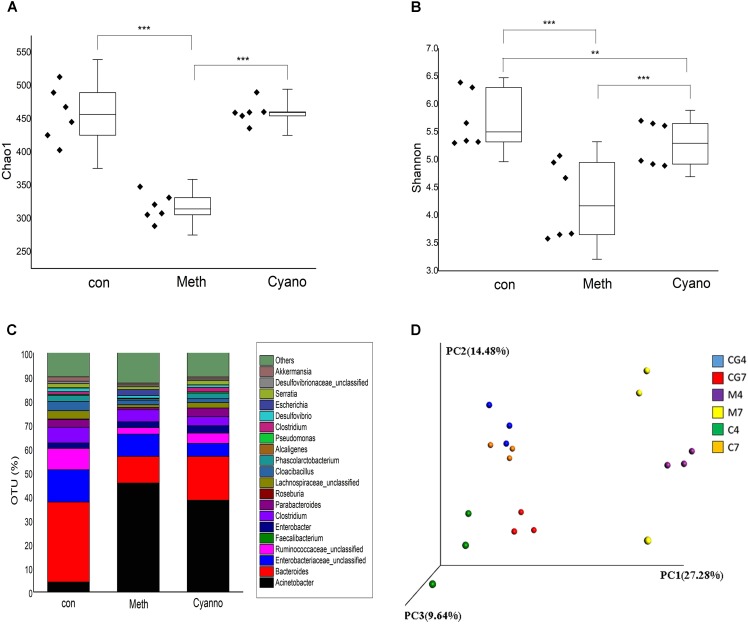
Alpha diversity of three groups via index Chao1 as **A** and Shannon as **B** (^∗^*p* < 0.05, ^∗∗^*p* < 0.001, and ^∗∗∗^*p* < 0.0001); Effect of various cobalamin on bacterial genera alternation in **C**. *con*, *Meth*, and *Cyano* stand for the control, Methylcobalamin, and cyanocobalamin groups, respectively; Principal component analysis (PCA) as **D** scores plot based on the relative abundance of OTUs (97% similarity level). Each symbol represents a sample, “4” means the fermented sample on the 4th day of the 2nd stage, “7” means the fermented sample on the 7th day of the 2nd stage.

A total of 12 samples from three groups supplemented with different types of cobalamin were chosen for microorganism profile analyses. After quality filtering, the 16S rRNA sequencing results produced 352,403 reads, giving a mean sample depth of 19,577.9 reads with a standard deviation of 9,002.1 reads. A total of 2,422 OTUs were obtained, and the relative abundance of OTUs showed the genera relative abundances in the samples. The alpha diversity of the microorganisms in the *Meth* group was significantly lower than those of the *con* and *Cyano* groups (Figure 2A) according to the Chao1 index. Based on the Shannon index, a higher diversity was found in the *Meth* group (Figure 2B). In terms of species homogeneity, both methylcobalamin and cyanocobalamin reduced the homogeneity of the types of microbes in the colon.

**FIGURE 3:**
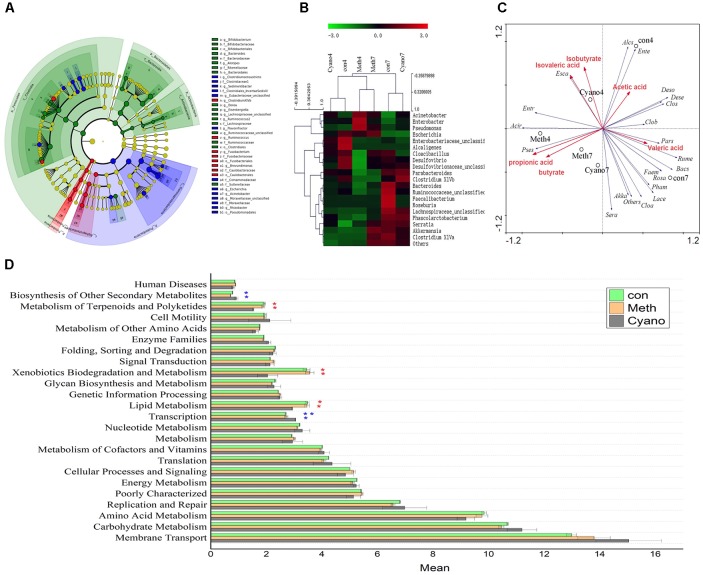
**(A)** Analysis of taxonomic abundances using LEfSe indicates that multiple taxa are differentially enriched in the three groups (The red color indicate *Cyano* group, the green color indicate con group, the blue color indicate con group). **(B)** Heatmap and hierarchical clustering (Euclidean distance) on the taxonomic genus level. **(C)** Canonical correspondence analysis (“Acir” means *Acinetobacter*, “Bacs” means *Bacteroides*, “Ente” means *Enterobacteriaceae*, “Rume” means *Ruminococcaceae*, “Faem” means *Faecalibacterium*, “Entr” means *Enterobacter*, “Cloa” means *Clostridium* XlVa, “Pars” means *Parabacteroides*, “Rosa” means *Roseburia*, “Lace” means *Lachnospiraceae*, “Clos” means *Cloacibacillus*, “Pham” means *Phascolarctobacterium*, “Alcs” means *Alcaligenes*, “Pses” means *Pseudomonas*, “Clob” means *Clostridium* XlVb, “Deso” means *Desulfovibrio*, “Esca” means *Escherichia*, “Sera” means *Serratia*, “Dese” means *Desulfovibrionaceae*, “Akka” means *Akkermansia*) **(D)** Comparisons of the predominant gene pathways of the microbiota in three groups.

Thirty-five different genera showed significant differences in relative abundances among the samples after 7 days of cobalamin supplementation (Figures 3A,B). As shown in Figure 2C, we found that 90% of the bacteria in the *in vitro* simulation were from 21 genera, including *Acinetobacter, Bacteroides, Enterobacteriaceae (unclassified), Roseburia, Ruminococcaceae (unclassified)*, and *Cloacibacillus*. However, after supplementation of methylcobalamin or cyanocobalamin, the microbiota in the samples (on the 7th day of the 2nd stage) had significantly shifted. Under *Meth* and *Cyano* conditions, the percentage of *Acinetobacter* spp. was increased to (45.54 and 31.86%, respectively), while the percentages of *Bacteroides* spp., *Enterobacteriaceae unclassified* spp., and *Ruminococcaceae unclassified* spp. had declined to 11.15 and 18.36%, 9.34 and 5.44%, and 2.69 and 4.31%, respectively. As shown in Figure 3A, *Cyano* (red) significantly increased *Clostridium XIVb* and *Fusobacterium* bacteria, while *Meth* (blue) increased the relative abundances of *Escherichia*, *Rhizobacter*, *Clostridium*, *Sedimentibacter*, and *Flavonifractor* bacteria. According to the results of hierarchical clustering (Figure 3B), the ecosystem structures of the *con* and *Cyano* groups exhibited high similarity, indicating that the microbial community structure was significantly influenced by *Meth*.

The first principal component accounted for 27.8% of the variance, the second principal component accounted for 14.8% of the variance, and the third principal component accounted for 9.64% of the variance. As shown in the PCA results (Figure 2D), there were few intragroup differences in the samples from the various groups except in the *Meth* group on the 7th day. However, samples from the different fermentation days exhibited large distinctions. The PCA results demonstrated that supplementation with cobalamin changed the bacterial composition in the colon simulation.

### Effects of the Various Cobalamins on SCFAs and Enzyme Activity

**FIGURE 4:**
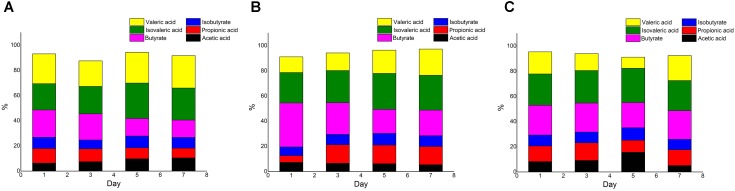
SCFAs composition ratio in *con* **(A)**, *Meth* **(B)**, and *Cyano* **(C)** groups during the *in vitro* colonic simulation.

The concentrations of total acid in the *Meth* and *Cyano* groups were higher than those in the *con* group (data not shown). The proportion of acetic acid increased during the experiment in the *con* group, while the proportion of propionic acid decreased. A similar trend was observed for the proportion of butyrate in the *con* group (Figure 4A). In the *Meth* group (Figure 4B), butyric acid accounted for the highest proportion on the first day of simulation, and the proportion was much higher than that in the other two groups. Then, the proportion of butyric acid gradually decreased over the next several days. Propionic acid represented the smallest proportion on the first day. The proportion of propionic acid rose rapidly and stabilized in the late middle stage of fermentation. In the *Cyano* group (Figure 4C), the proportions of each SCFA did not change.

**FIGURE 5:**
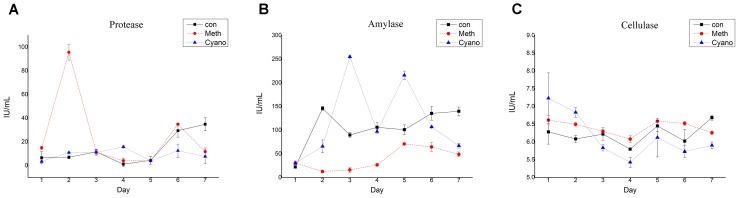
Shifts of protease **(A)**, amylase **(B)**, and cellulase **(C)** activity during simulation with/without *Meth*, and *Cyano* supplementation. One protease activity unit (U) was defined as quantities of enzyme in 1mL enzyme solution, which cleaved casein to produce 1 μg tyrosine per minutes at 40°C in a condition with pH value of 7.0. One amylase activity unit (U) was defined as quantities of enzyme in 1 mL enzyme solution, which degraded 10 mg starch for 30 min at 40°C. One unit of cellulase activity (U) was defined by quantities of enzyme in 1 mL enzyme solution, which hydrolyzed carboxymethyl cellulose to 1 mg glucose in 30 min.

The protease activity in the *con* and *Cyano* groups was maintained at a low level during the first 5 days (Figure 5A). The protease activity in the *con* group increased over the last 2 days to levels significantly higher than those in the *Cyano* group. The protease activity in the *Meth* group rose rapidly from 14.87 ± 1.56 U/g to 95.54 ± 6.53 U/g on the 2nd day and then decreased rapidly (Figure 5A). This phenomenon can be illustrated by the rapid change in SCFAs in the *Meth* group on the first day. The amylase activity in the three groups fluctuated in the range of 5.5 to 7.2 U/g during the simulations (Figure 5B). The amylase activity was more stable in the *con* group than in the other two groups. A statistically significant difference was found in cellulase activity among the *con*, *Cyano* and *Meth* groups (Figure 5C) from the 3rd to the 5th day. Regarding the enzyme carboxymethyl cellulase, the activity of cellulase in the *Cyano* group was the most unstable and fluctuated in a wide range from 24 U/g to 256 U/g. In addition, the cellulase activity in the *Meth* group was observed to be lower than that in the *con* and *Cyano* groups.

### Relationship Between Bacteria and SCFAs as Determined by Canonical Correspondence Analysis

The distance between two points showed the similarity in the community structures of two samples. An acute angle between two factors indicated that the two factors were positively correlated. In contrast, an obtuse angle exhibited a negative correlation. The effects of the factors were shown by the lengths. As shown in Figure 3C, we found that *Pseudomonas* spp. were positively correlated with the yield of propionic acid and butyrate in the simulations. In addition, *Meth* was positively correlated with *Pseudomonas* bacteria, propionic acid, and butyrate. The amount of *Meth* supplementation increased the relative abundance of *Pseudomonas* bacteria and promoted the production of propionic acid and butyric acid. *Acinetobacter*, *Enterobacter*, and *Escherichia* bacteria were positively correlated with isovaleric acid and isobutyric acid content. Bacteria such as *Ruminococcaceae* and *Parabacteroides* spp. were positively correlated with valeric acid content, and *Desulfovibrio* and other genera were positively correlated with acetic acid content.

### Function Prediction of Gut Microbiota via PICRUSt

Fourteen primary KEGG (Kyoto Encyclopedia of Genes and Genomes) metabolic pathways, 24 secondary KEGG metabolic pathways, and 262 tertiary KEGG metabolic pathways were obtained through PICRUSt prediction. The majority of secondary metabolic pathways were not affected by cobalamin. Cobalamin promoted lipid, terpenoid, and polyketide metabolism by gut bacteria, promoted the degradation of exogenous substances, and inhibited the synthesis of transcription factors and secondary metabolites. Among the tertiary metabolic pathways (Figure 3D), the DNA repair and recombinant protein (ko03400) pathway was increased significantly in the cobalamin groups. Nitrogen metabolism (ko00910) and starch with sucrose metabolism (ko00500) declined in the *Meth* and *Cyano* groups. During *Meth* and *Cyano* supplementation, the expression of the ABC transporter (ko02010) pathway increased significantly in the *con* group. Both forms of cobalamin had a similar influence on the metabolism of the microbiota.

## Discussion

In this study, the gut flora from cobalamin deficiency patients were dramatically changed after methylcobalamin and cyanocobalamin supplementation. Some beneficial bacteria, such as *Faecalibacterium prausnitzii* and *Roseburia* spp. with abilities to produce butyrate and alleviate inflammation in gastrointestinal tract, were reduced in the cobalamin groups. In our work, we found that *Meth* and *Cyano* increased the relative abundance of *Proteobacteria* spp. (46.70 and 35.64%, respectively) and decreased the relative abundances of *Firmicutes* (25.67 and 22.96%, respectively) and *Bacteroidetes* (20.45 and 10.52%, respectively) bacteria. Unfortunately, the changes in the gut microbiota after *Meth* and *Cyano* supplementation were similar to those in the gut microbiota of patients with necrotizing enterocolitis, which increases the relative abundance of *Proteobacteria* spp. and decreases the relative abundance of *Firmicutes* and *Bacteroidetes* spp. (Pammi et al., 2017). Miyake et al. (2015) also stated that the relative abundance of *Prevotella* spp. and *Firmicute*s bacteria such as *Faecalibacterium* spp. is reduced in the fecal microbiota of patients with multiple sclerosis. Therefore, supplementation with high concentrations of *Cyano* in patients should be carefully advised and regulated.

In this study, we found that *Meth* and *Cyano* not only reduced the total bacteria but also reduced the diversity and homogeneity of colon flora. These findings indicate that cobalamin has a selective effect on the intestinal microflora. Previous studies have found that hemoglobin, which also has a porphyrin ring structure, has the same effect. Constante et al. (2017) found that dietary haem also reduced the diversity of fecal flora in mice. Substances with porphyrin ring structures may act as modulators of the intestinal flora. In our work, we found that both forms of cobalamin enhanced the relative abundance of *Acinetobacter* spp., especially in the *Meth* groups, in which the relative abundance was up to 47.54%. After searching the NCBI database, we found several genes related to cobalamin-dependent methionine synthase in *Acinetobacter* spp., indicating that methylcobalamin is an essential growth factor for *Acinetobacter* spp.

Some researchers have reported that *Acinetobacter* spp. are frequently found and isolated in nosocomial infections, which are especially prevalent in intensive care units. *Acinetobacter* spp. have been implicated in a great number of hospital-acquired infections, such as wound and burn infections, secondary meningitis, urinary tract infections, infective endocarditis, and bacteraemia (Dent et al., 2010). Some *Acinetobacter* spp. have the capacity to develop resistance to many available antibiotics and to transfer these plasmids to other pathogenic bacteria by horizontal gene transfer (Shamsizadeh et al., 2017). Due to the high risk of *Acinetobacter* spp. infection, we suggest that patients with cobalamin deficiency be supplemented with cyanocobalamin, which did not enhance the relative abundance of *Acinetobacter* spp. as much as did methylcobalamin.

The results of this experiment showed that cyanocobalamin improved the relative abundances of *Fusobacteriaceae*, *Ruminococcus*, and *Brevundimonas* bacteria, which are associated with inflammation and cancer, but that methylcobalamin enhanced the growth of butyrate-producing bacteria, suggesting that methylcobalamin may alleviate intestinal inflammation. Many studies have shown that vitamin B12 deficiency is associated with inflammatory bowel disease (Headstrom et al., 2008; Pawlak, 2015). Wang et al. (2007) tried to use lamb stomach extract cobalamin capsules to treat elderly patients with diarrhea. This study can act as a guide for the development of cobalamin treatments to improve intestinal flora and treat intestinal diseases.

The results of this study showed that cobalamin increased the total SCFA content and adjusted the SCFA composition. SCFAs are mainly composed of acetic acid, propionic acid, and butyric acid derived from carbohydrate metabolism by intestinal bacteria. Isobutyric acid and isovaleric acid are branched-chain SCFAs derived from the decomposition of proteins. Absorption of acetic acid can increase cholesterol synthesis in the human body, while propionic acid can inhibit cholesterol synthesis. Therefore, the risk of cardiovascular disease can be reduced by balancing the ratio of acetic acid to propionic acid (Wong et al., 2006). Butyric acid is the key source of energy for colon cells; it can also promote cell growth and differentiation, nourishing the intestinal mucosa and playing a role in the prevention of colon cancer. In our study, we found that methylcobalamin reduced the ratio of acetic acid to propionic acid and increased the proportion of butyrate, but cyanocobalamin and the control treatment did not cause large shifts. Zhao and Meng (2006) also reported that the amount of total volatile acid exhibited an upward trend with increasing B12 supplementation concentrations. The production of acetic acid in the 30 and 90 ng/mL groups increased by 4.97 and 5.76%, respectively, and the production of propionic acid increased by 5.31 and 6.4%; these increases are lower than those in our study. Because cobalamin plays an important role in the metabolism of lipids, carbohydrates, and proteins, promoting the metabolic rate of gut microbiota, the total SCFA content was increased with the addition of cobalamin. The interaction among the three factors (gut microbiota, short-chain fatty acids, and cobalamin) were also calculated in our study. Studies have found that the content of branched-chain short-chain fatty acids is positively correlated with the abundance of *Escherichia* bacteria. According to these results, methylcobalamin supplementation should be suggested to enhance the production of propionic acid and butyrate.

Intestinal flora can produce a large number of enzymes, mainly including β-glucosidase, β-glucuronidase, nitroreductase, protease, and various carbohydrate enzymes (Chen et al., 2014). Different types of bacteria can produce different enzymes that can catalyze different types of reactions. Some of these metabolic enzymes can help the host hydrolyse drugs so that the drugs can exert their effects, and some are harmful to the host. Intestinal metabolic enzymes are also associated with host inflammation (Bischoff, 2017). Li (2012) reported that the addition of cobalamin significantly increased the activity of carboxymethyl cellulase and amylase in the rumen. These result are different from the results obtained in our *in vitro* human colon simulation. We found that cobalamin did not increase amylase or protease activity in the fermentation broth, but the effects of the different forms of cobalamin on carboxymethyl cellulase showed different trends. The relative abundance of *Pseudomonas* bacteria, which have a strong ability to produce carboxymethyl cellulase, was increase in the *Meth* group, but the cellulase activity in that group was only increased on the 2nd day. Unexpectedly, no large differences in protease activity were observed under the different cobalamin conditions. In this study, the expression of the ABC transporter (ko02010) pathway in the cobalamin groups was higher than that in the control group. Bertrand et al. (2012) found that in a low-B12 environment, algae can increase the expression of B12 transporters to take in more B12, which is inconsistent with our results. As reported previously, cobalamin riboswitches are located in 5′-untranslated regions of genes related to cobalamin and can respond to cobalamin concentrations (Mellin et al., 2013). Under high concentrations of B12, the B12 riboswitch of btuB in *E. coli* and *Salmonella typhimurium* (Nahvi et al., 2004) bound to B12 and shut off the expression of downstream genes. Various forms of cobalamin have the ability to mediate some gut bacteria by switching riboswitches on or off to balance energy and other resources.

The various cobalamin applications in the food industry should be monitored and regulated, because the two types of cobalamin had different effects on the gut microbiome and on microbial metabolism, although they have equal bio-activity in humans. In this study, we found that methylcobalamin and cyanocobalamin had completely different effects on bacterial composition, gut microbiome diversity, SCFAs, and enzymes in an *in vitro* colon simulation. We also demonstrated that methylcobalamin can promote the growth of *Pseudomonas*, *Acinetobacter*, and butyrate-producing bacteria in an *in vitro* colon simulation to balance the intestinal environment and that cyanocobalamin can also reduce the relative amount of *Clostridium* to reduce inflammation in intestinal environment. In addition, methylcobalamin can enhance the production of SCFAs, especially butyrate, to maintain the intestinal environment. Moreover, methylcobalamin promoted lipid, terpenoid and polyketide metabolism by gut bacteria, promoted the degradation of exogenous substances, and inhibited the synthesis of transcription factors and other secondary metabolites. Given the benefits of methylcobalamin on the gut microbiota and on microbial metabolism, methylcobalamin supplementation should be suggested as the first option.

## Author Contributions

YX, SX, and XZ contributed to the design, organization, and conduction of the whole experiments. KY, YZ, and XF contributed to the statistics and prediction. JC and YC contributed to SCFA analysis experiments.

## Conflict of Interest Statement

The authors declare that the research was conducted in the absence of any commercial or financial relationships that could be construed as a potential conflict of interest.

## Footnotes

1 http://qiime.org/tutorials/processing_illumina_data.html

2 https://github.com/torognes/vsearch, v2.3.4

3 http://ccb.jhu.edu/software/FLASH

4 http://huttenhower.sph.harvard.edu/galaxy/
